# Next-generation sequencing identified a novel *DYSF* variant in a patient with limb-girdle muscular dystrophy type 2B: A case report

**DOI:** 10.1097/MD.0000000000022615

**Published:** 2020-10-09

**Authors:** Qiao Li, Cheng Tan, Jiajun Chen, Lei Zhang

**Affiliations:** Department of Neurology, China-Japan Union Hospital of Jilin University, Changchun, Jilin, China.

**Keywords:** dysferlin gene, limb-girdle muscular dystrophy type 2B, muscle biopsy, next-generation sequencing

## Abstract

**Rationale:**

Limb-girdle muscular dystrophy (LGMD) is a genetic disease, which is characterized by muscle atrophy and weakness mainly involving proximal muscles. Accurate diagnosis of LGMD patient is very important for the appropriate management and long-term prognosis.

**Patient concerns:**

An 18-year-old woman presented with progressive weakness of limbs, persistent elevated serum creatine kinase, myogenic damages in electromyography, and dysferlin protein deficiency in muscle biopsy. Further next-generation sequencing (NGS) revealed a compound heterozygous variant in dysferlin gene (*DYSF*), including a novel frameshift variant of c.4010delT.

**Diagnosis:**

The patient was diagnosed with LGMD2B clinically and genetically.

**Interventions:**

Oral levocarnitine and coenzyme Q10 were prescribed to the patient.

**Outcomes:**

After symptomatic treatments for 1 week, the patient's symptoms were not improved.

**Lessons:**

NGS might be a helpful tool for the diagnosis of LGMD. A novel variant of c.4010delT in *DYSF* was identified in this case, which broadens the genetic spectrum of LGMD2B.

## 1 Introduction

Limb-girdle muscular dystrophy (LGMD) encompasses a group of progressive muscular disorders, which present with muscular weakness and atrophy in predominantly proximal muscles. It can be divided into LGMD type1 and LGMD type 2 according to genetic pattern.^[1]^ Accurate and prompt diagnosis of LGMD including genetic diagnosis is very necessary and important not only for the appropriate management, long-term prognosis, but also for the genetic counseling and the future possible gene therapy.^[2]^ Here, we reported a woman complaining of the weakness of limbs. Further examinations showed persistent elevated serum creatine kinase (CK), myogenic damages in electromyography (EMG), and dysferlin protein deficiency in muscle biopsy. Next-generation sequencing (NGS) revealed a compound heterozygous variant in the gene encoding dysferlin (*DYSF*), including a novel frameshift variant of c.4010delT. These results broadened the spectrum of LGMD2B and indicated that NGS might be a powerful tool for the diagnosis of LGMD.

## 2 Clinical data

**Figure 1:**
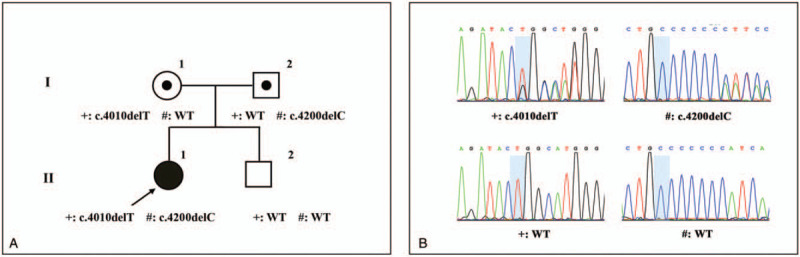
The sequencing results of *DYSF* in the pedigree. (A) The pedigree of the family compiled with autosomal recessive inheritance. The symbols “+” and “#” indicate a carrier of the variant described behind. WT means wild-type genotype. (B) Sanger sequencing results of *DYSF*. The left-hand column shows the novel frameshift variant of c.4010delT and the wild-type genotype. The right-hand column shows the pathogenic frameshift mutation of c.4200delC and the wild-type genotype. The blue background highlights the site of the variant.

An 18-year-old woman (II-1 in Fig. 1A) came to our hospital in June 2018. She presented with a 5-year history of insidiously progressive weakness of lower limbs and 2-month weakness of upper limbs. At the age of 13, she noticed *symmetrical* proximal weakness of both lower limbs. The symptom was obvious when she tried to climb the stairs or stand up after squatting. She did not complain of any weakness in the upper limbs then. The weakness of lower limbs worsened gradually. Two months before she came to our hospital, she felt weakness of upper limbs when she held heavy objects, but could comb, wash face, and use chopsticks normally. When she came to our hospital, her walking speed was slow and she has some difficulty walking upstairs. She never complained of dysphagia, myalgia, or other clinical manifestations. She underwent multiple examinations for muscle enzyme. Serum CK was consistently elevated, ranging from 7845 to 37,906 IU/L (normal range 26–140 IU/L). She did not receive any specific treatment except irregular oral intake of Vitamin B1 and B12. The patient denied consanguineous marriage or genetic diseases in the family. This study has been reviewed and approved by the Ethics Committee of the China-Japan Union Hospital of Jilin University. The patient and her family have provided written informed consent to the participation in the study and authorized to publish the study in accordance with the Declaration of Helsinki.

### 2.1 Physical examination

The patient had difficulty in climbing upstairs or rising independently after squatting. She could not stand on her tiptoes or heel and had a wadding gait. Physical examination revealed bilateral symmetrical muscle weakness, proximal more severely than distal. She had no fasciculations or myotonia with a normal muscular tone. She had areflexia in bilateral knee tendon. The biceps and triceps tendon reflexes were hyporeflexia. Babinski sign was negative bilaterally.

### 2.2 Laboratory investigation

Comprehensive infectious, metabolic, paraneoplastic, and inflammatory examinations of the patient were negative. Serum CK was found markedly elevated to 4645 U/L (normal range 26–140 IU/L). Serum CKs of her parents were also tested and were found normal (76.89 and 63.99 IU/L for her father and mother, respectively).

### 2.3 EMG and muscle imaging

The nerve conduction tests presented normal. The needle EMG test showed small polyphasic motor units potentials of rapid recruitment in biceps femoris, triceps surae, rectus femoris, and internal femoral muscle, indicating a myopathic process.

**Figure 2:**
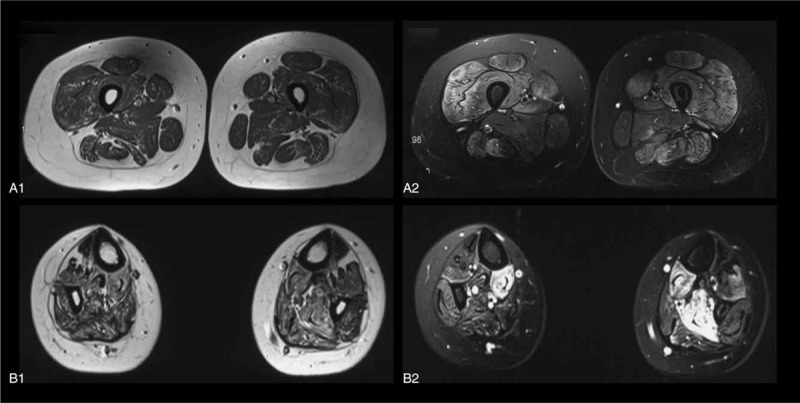
Transverse MRI images of the thighs and calves show fatty infiltration and atrophy of the muscles. The images are T1-weighted (left-hand column) and STIR sequence (right-hand column).

Magnetic resonance imaging (MRI) of lower limbs showed fatty infiltration and atrophy of the thigh and calf muscles, presenting as the high signal in T1-weighted and short-time inversion recovery (STIR)-weighted images (Fig. 2).

### 2.4 Muscle pathology

Histopathology of left quadriceps exhibited an increased variation of fiber size with mild fatty infiltration indicating a dystrophic process. At the same time, endomysial fibrosis could also be seen. Immunohistochemistry analysis showed a complete dysferlin deficiency in biopsied muscle.

### 2.5 Genetic testing

Targeted NGS was performed on the patient's DNA for 200 genes currently related to muscular disorders in Beijing Running Gene Corporation. A compound heterozygous variant of c.4010delT and c.4200delC in *DYSF* was found. This genetic result was further confirmed by direct Sanger sequencing (Fig. 1B). The frameshift variant of c.4010delT was a novel one according to Leiden Muscular Dystrophy database (www.dmd.nl, updated January 29, 2020). It was located in exon 38 of *DYSF* and predicted inducing p.L1337Rfs∗8. According to American College of Medical Genetics and Genomics standards and guidelines,^[3]^ this variant was classified as “likely pathogenic.” The patient also harbored the pathogenic mutation of c.4200delC in exon 39, which was predicted inducing p.I1401Sfs∗47.^[4–6]^ The patient inherited these 2 variants from her mother and father, respectively. Her brother carried wild-type alleles (Fig. 1A and B).

### 2.6 Treatment

Oral levocarnitine and coenzyme Q10 were prescribed to the patient for 1 week, but the symptoms of weakness were not improved.

## 3 Discussion

Dysferlinopathies encompass a heterogeneous group of muscular disorders, which are characterized by the absence of dysferlin in skeletal muscles. The mutations in *DYSF* (MIM∗603009) cause these autosomal recessive diseases. So far, there are 3 main phenotypes in dysferlinopathies, that is, Miyoshi myopathy, LGMD 2B, and distal myopathy with anterior tibial onset. The prominent clinical presentations of LGMD2B are weakness and atrophy of the pelvic and shoulder girdle muscles. Onset is early, typically in adolescence or young adulthood. At late-stage, muscle bulk in the pelvic girdle and calf may lose, resulting in falls down and difficulty in walking upstairs or running. In most patients, there is no early cardiac or respiratory involvement. Massive elevated serum CK indicates a muscular injury. Typically, it is 10 to 150 times above the normal level. ^[7,8]^ This patient suffered from chronic progressive symmetric aggravation of proximal muscle weakness with insidious onset. The weakness first involved in lower limbs later upper limbs. Consistent elevated CK level and EMG results indicated muscle-derived injury. Leg MRI showed widely spread muscular dystrophy. Immunohistochemical analysis on muscle biopsy indicated an absence of dysferlin, conforming to dysferlinopathy. On the basis of the evidences above, the clinical diagnosis of LGMD2B was made.

However, sometimes, the diagnosis of LGMD 2B is challenging. The clinical presentations might be atypical. Furthermore, a secondary dysferlin deficiency may also be observed in other myopathies such as calpainopathy, sarcoglycanopathy, and dystrophinopathy.^[6,8–10]^ So, further genetic testing to identify the pathogenic mutations in *DYSF* is very important for accurate diagnosis and to rule out other differential diseases.^[8]^ However, Sanger sequencing is the golden method without a doubt, but it is time-consuming and labor-intensive for *DYSF* gene because of its large size (6243 base-pairs and 55 coding exons, spanning a genomic region of over 233 kb) and the absence of mutational hot spots.^[11]^ NGS is an alternative approach because it is much more efficient and cost-effective, especially in children and in those patients with mild, nonspecific, or atypical phenotypes.^[6,12,13]^ So, NGS has been suggested as a last resort after clinical examination and identification of geographic/ethnic origin in the American Academy of Neurology guidelines for LGMD.^[2]^ In this case, NGS identified a compound heterozygous frameshift variant in *DYSF* and confirmed her final diagnosis of LGMD2B. In these 2 variants found, c.4010delT was a novel one. It was classified “likely pathogenic” according to American College of Medical Genetics and Genomics standards and guidelines. ^[3]^ It confirmed the clinical diagnosis of the patient and broadened the genetic spectrum of LGMD2B.

The current treatment to LGMD2B is supportive whose principle is to maintain mobility and functional independence as long as possible. It includes physical therapy, mechanical aids, respiratory aids, surgical intervention for orthopedic complications and social support. Physical trauma, bone fractures, immobility, high-intensity exercises, and obesity should be avoided for optimizing muscle strength and function. Regular physiotherapy is helpful to prevent skeletal deformities, scoliosis, and contractures. If the patient has developed musculoskeletal spine deformities, an orthopedic spine surgeon should monitor and consider surgical intervention if necessary. Gentle, low-impact “aerobic exercise such as swimming and stationary bicycling can benefit the patients and improve cardiovascular performance. Mechanical aids such as canes, walkers, orthotics, and wheelchairs are helpful for the late-stage patients to keep ambulating. The effect of other treatment, including exon-skipping, viral gene therapy, stem cell transplantation, neutralizing antibody to myostatin, or growth hormone are yet to be investigated.^[2]^ Further studies are ongoing to explore possible therapies for LGMD2B.

In this case, the novel variant of c.4010delT was considered the cause of LGMD2B according to the patient's clinical presentations and the American College of Medical Genetics and Genomics standards and guidelines. However, further study on this variant in vivo and in vitro is necessary to clarify its pathogenicity and pathogenesis, and will help the exploration of new treatment strategy. Furthermore, the frequency of c.4010delT should be investigated in different population for an optimal genetic testing strategy.

## Author contributions

**Methodology:** Qiao Li, Cheng Tan.

**Supervision:** Lei Zhang.

**Writing – original draft:** Qiao Li, Cheng Tan.

**Writing – review & editing:** Jiajun Chen.

## Abbreviations

Abbreviations: CK = creatine kinase, *DYSF* = dysferlin gene, EMG = electromyography, LGMD = limb-girdle muscular dystrophy, MRI = magnetic resonance imaging, NGS = next-generation sequencing, STIR = short-time inversion recovery.
